# Choroidal Thickness in Women with Uncomplicated Pregnancy: Literature Review

**DOI:** 10.1155/2017/5694235

**Published:** 2017-11-09

**Authors:** Joanna Roskal-Wałek, Iwona Laudańska-Olszewska, Michał Biskup, Magdalena Gierada, Dominik Odrobina

**Affiliations:** ^1^Department of Ophthalmology, Voivodeship Regional Hospital, Kielce, Poland; ^2^Ophthalmology Clinic of St. John Boni Fratres Lodziensis, Łódź, Poland; ^3^Faculty of Health Sciences, Jan Kochanowski Memorial University of Kielce, Kielce, Poland

## Abstract

Pregnancy is a time when many changes occur in a woman's body. The goal of these changes is the provision of optimum conditions for the development of the foetus. Pregnancy also affects eye physiology. Well recognized physiological changes include a reduced corneal sensitivity, an increase in its central thickness and curvature, and a decrease in intraocular pressure. The association between choroidal thickness and pregnancy is not clear. Haemodynamic and hormonal changes taking place during pregnancy and the question of whether these changes are reflected by choroidal thickness are especially important. It is assumed that the choroid, which is one of the most highly vascularized tissues characterized by the highest blood flow to tissue volume ratio in the whole body, should respond by an increase in its thickness to an increase in blood flow and drop in the value of peripheral resistance. Measurement of choroidal thickness using enhanced depth imaging optical coherence tomography (EDI-OCT) in women with uncomplicated pregnancy provides important information concerning the effects of physiological changes in the eye.

## 1. Introduction

Pregnancy is a time when many changes occur in a woman's body, the primary goal of which is the provision of proper conditions for the development of the foetus. Metabolic, haematological, vascular, hormonal, and immunological changes lead to the necessary anatomical and functional adaptive changes [1, 2].

The changes taking place during pregnancy affect also the eye [1, 3]. Most changes, such as an increase in the central corneal thickness (CCT), change in its curvature, decrease in corneal sensitivity, or a decrease in intraocular pressure (IOP) are physiological and transitional. Under the effect of hormonal changes some disorders may become aggravated, such as diabetic retinopathy, for example. Pathological conditions, such as arterial hypertension or preeclampsia may complicate the course of pregnancy and lead to the development of ocular changes [1, 3].

From the beginning of pregnancy, the cardiovascular system of the pregnant woman is subject to especially important changes. During pregnancy, the cardiac output and heart rate increase. As early as the first trimester of pregnancy, due to an increase in the renin-angiotensin-aldosterone system, an increase in the blood volume occurs. Hormonal changes lead to dilatation of the blood vessels and a reduction in systemic vascular resistance, which is accompanied by an increase in vascular capacity. Blood flow in many organs increases [2].

The choroid, which is one of the most highly vascularized tissues characterized by the highest blood flow to tissue volume ratio in the whole body, may be especially susceptible to haemodynamic and hormonal changes taking place in pregnancy, which may affect its thickness [4, 5].

Before the introduction of enhanced depth imaging optical coherence tomography (EDI-OCT) examinations, the possibilities of assessment of the choroid were considerably limited, with respect to both physiological and pathological changes. Ultrasonography, fluorescein angiography, and indocyanine angiography, specially dedicated to the evaluation of choroidal circulation, did not provide such diagnostic possibilities as those presently offered by the EDI-OCT examination [6, 7]. In addition, angiography, due to its invasive character, is limited in its use as an examination for pregnant women because of concerns about possible complications for the mother and foetus [8]. The EDI-OCT examination is noninvasive, simple, and quick. This method makes it possible to precisely evaluate the choroid, including the measurement of its thickness, which may be influenced by both physiological and pathological factors [6, 7, 9].

The objective of this study was a review of the present literature concerning choroidal thickness in EDI-OCT imaging in women with an uncomplicated course of pregnancy.

## 2. Discussion

### 2.1. Choroidal Blood Flow

The choroid lying between the retina and the sclera is built mainly of blood vessels and constitutes the main source supplying blood and nutrients to the outer retina. Also, the choroid performs a thermoregulation function, moves the location of the retina due to changes in its thickness, and is responsible for the secretion of growth factors [4]. Although the basic functions of the choroid are known, the relationship between the choroidal thickness and blood flow in the eyeball has not been fully recognized and evokes great interest. The potential relationship between choroidal thickness and choroidal circulation has been investigated many times [10–13]. Many techniques have been developed to monitor choroidal blood flow; nevertheless, these measurements are especially difficult because the choroidal vessels are “hidden” beneath the retinal pigment epithelium, and blood flow is assessed mainly indirectly [14]. The studies conducted to-date concerning blood flow in the eye in pregnant women presented contradictory results [5, 15–17]. For a long time, it has been assumed that, in contrast to the retina and the anterior part of the vascular membrane, the choroid does not show evidence of autoregulation and is mainly subject to the effect of the autonomic nervous system and circulating hormones [4, 18]. It was suggested that the prevalence of estrogen leads to dilation of the vascular bed and an increase in blood flow through the choroid [5]. Recent studies show the presence of some degree of autoregulation also on the level of choroidal blood flow [11, 12, 14]. The process of choroidal autoregulation participating in the control of blood flow seems to be very complex and has been poorly recognized to-date [14].

To assess whether an increase in choroidal thickness occurs during pregnancy in response to the ongoing haemodynamic and hormonal changes, choroidal thickness was examined using a new imaging technique, EDI-OCT [9, 19–26]. The heretofore choroidal imaging by means of spectral-domain optical coherence tomography (SD-OCT) was limited, mainly due to the difficulties with transmission of the signal through the retinal pigment epithelium. The EDI-OCT, which is the modification of the standard OCT, provides resolution to approximately 3-4 microns and deeper scanning, which allows visualization and dimensioning of the choroid [27, 28].

### 2.2. Factors Affecting Choroidal Thickness

Studies comparing choroidal thickness in nonpregnant and pregnant women provide contradictory results. According to some studies, a statistically significant thickening of the choroid occurs during pregnancy [9, 19, 21–26, 29], whereas other studies do not show such a relationship [20, 26]. Also, the results concerning the relationship between choroidal thickness and gestational age differ [9, 20–25]. Some of the available studies evaluate not only the choroidal thickness in women with an uncomplicated pregnancy, but also choroidal thickness in pregnancy complicated by preeclampsia. These results differ with respect to both choroidal thickness in pregnant women and the thickness of the choroid in women with preeclampsia, compared to choroidal thickness in nonpregnant women [19, 24, 26]. The differences also occur while comparing choroidal thickness between pregnant women and those with preeclampsia [19, 24, 26].

The variability of results may be caused by many factors. Studies differ with respect to such variables as number of patients examined, patients' age, gestational age at performing the examination, and differences occurring with respect to the refraction interval within which the women are enrolled into the study; these differences are relatively big for emmetropia as well -6D [20, 23]. All these factors may exert an effect on the results obtained. Many studies confirmed the effect of such factors as age, gender, axial length of the eye (AL), refraction defect, CCT, or the IOP level of the choroidal thickness [30–32]. A comprehensive study concerning the evaluation of the subfoveal choroidal thickness (SFCT) which covered 3,233 patients showed that the SFCT is higher in younger individuals, in shorter eyeballs, in males, in eyes with a deeper anterior chamber, thicker lens, flat retina, and better best corrected visual acuity (BCVA). This study indicates that these factors should be considered while performing measurements of the choroidal thickness [33]. In most of the studies available, the researchers refer to women's age, CCT, AL, IOP, and refraction defect, and assess the effect of these factors on the choroidal thickness (Table 2 presents the effect of these factors on choroidal thickness according to the studies available).

The differences between available studies also occur with respect to the equipment applied for the examination. Most examinations were performed using the Heidelberg Spectralis and Cirrus-OCT. However, the study by Koay et al. did not show any statistically significant difference between the results concerning choroidal thickness measured by means of such equipment as SD-OCT: Zeiss Cirrus HD-OCT, Heidelberg Spectralis, and Optovue RTVue [34]. The repeatability of the SFCT measurements using various machines was also confirmed in the study by Yamashita et al. [35]. Studies pertaining to choroidal thickness in pregnant women do not differ with respect to the method of performing the examinations; measurements were performed manually or automatically; the differences also concern the areas covered by the examination. In some studies only the SFCT is measured; in other studies measurements are performed in three areas of reference, that is, subfoveal, nasal, and temporal, within specified intervals, while single studies consider a larger number of areas using the protocol used in the Early Treatment Diabetic Retinopathy Study (ETDRS). At present, it is recommended that measurements of choroidal thickness be performed using the ETDRS protocol charts, which work best in an objective evaluation of the choroid considering its varied topography [36, 37]. Due to daily fluctuations in choroidal thickness, the time at which the examination is performed is also important, which should be the same in all studies, while in some studies this factor was not taken into consideration [38]. Iwase et al., in their study, found that the subfoveal choroidal thickness is subject to significant diurnal variations. It was observed that during the day the subfoveal choroidal thickness decreases and the lowest values of choroidal thickness were noted at 3 p.m., whereas at night an increase in the choroidal thickness was observed [38]. In the light of the results of these studies, the selection of the control group may also be important. Few studies consider the phase of the menstrual cycle of women in the control group, which is important considering the relationship confirmed between the effect of hormones and choroidal thickness [39]. Apart from the study by Dadaci et al. [23], none of the studies referred to the phase of the menstrual cycle in women from the control group. In the studies, there is neither information concerning the hormonal profile of the control group nor data concerning whether these women used hormonal contraception (Table 1 demonstrates the differences between studies).

### 2.3. Choroidal Thickness in Pregnant and Nonpregnant Women

Liu et al. performed meta-analysis in which, despite many differences occurring between the studies included in the meta-analysis, they found a statistically significant difference in the choroidal thickness between pregnant and nonpregnant women. The same relationship was also confirmed in their own study, where the choroidal thickness in pregnancy was statistically significantly higher [9].

Apart from the meta-analysis available, studies on the choroidal thickness concern small groups of examined women. The study in which the largest number of women were examined from the perspective of choroidal thickness was conducted by Kara et al., who compared choroidal thickness in 100 pregnant women (at week 15 and 38 of pregnancy) and 100 women who were not pregnant. The results indicated that the choroidal thickness was statistically significantly higher in pregnancy; however, a statistically insignificant relationship was noted between the choroidal thickness and gestational age [21]. Similarly, in the study by Ulusoy et al., statistically significant differences were confirmed between the choroidal thickness in pregnant and nonpregnant women. Also, in this study, no statistically significant difference was found between the choroidal thickness and gestational age [22]. This is the only study also evaluating choroidal thickness in the same patients after delivery. In the EDI-OCT examination performed three months after childbirth, a decrease was observed in the choroidal thickness, compared to the period of pregnancy [22]. Ataş et al., and Rothwell et al., in their studies, examined women in the third trimester of pregnancy, and also found that in these women the choroidal thickness was significantly higher statistically, compared to women who were not pregnant [19, 29].

Dadaci et al. compared choroidal thickness in healthy pregnant women in the first and third trimester of pregnancy. The study covered 27 (54 eyes) pregnant women and 25 women (50 eyes) who were not pregnant. Pregnant women had the EDI-OCT examination performed twice, in the first trimester, that is, weeks 6–8 of pregnancy, and subsequently in the third trimester, weeks 32–37, while the control group had the examination performed once in the follicular phase of the cycle. The choroidal thickness in all the measured areas, that is, subfoveal, nasal, and temporal, the foveal centre in 500 um intervals in both eyes was significantly lower statistically in the third trimester, compared to the thickness measured in the same areas in the first trimester. The choroidal thickness in nonpregnant women was lower; however, it was statistically insignificant [23]. Similarly, in the study by Sayin et al., a negative correlation was observed between choroidal thickness and gestational age (women in week 17–37 of pregnancy were enrolled in the study); however, various women were examined in the second and third trimester, and in this study a significantly higher statistically choroidal thickness was also found in pregnant women, compared to those who were not pregnant [24]. Similarly, Goktas et al. also enrolled various women at various gestational ages into their study [25]. This is the first study in which the choroidal thickness was compared between the three trimesters of pregnancy. The study covered 30 women in the first, 30 women in the second, and 30 women in the third trimester of pregnancy, as well as 30 healthy nonpregnant women. The analysis showed statistically significant differences between the choroidal thickness in the second trimester of pregnancy and choroidal thickness in women who were not pregnant. The researchers presume that the thickening of the choroid may occur in the subfoveal, nasal, and temporal areas in the second trimester [25].

### 2.4. Choroidal Thickness and Haemodynamic and Hormonal Changes During Pregnancy

Most researchers investigating choroidal thickness in pregnant women interpret changes in the choroidal thickness with reference to the haemodynamic and hormonal changes which occur in pregnancy [9, 19–26, 29].

During pregnancy, an increase in the blood volume, an increase in cardiac output caused by an increase in the stroke volume, and acceleration of the heart rate occur. A decrease in systemic resistance also occurs. Hormonal changes taking place in pregnancy are the cause of considerable haemodynamic changes. As early as the first weeks of pregnancy, changes in the hormonal balance are clearly observed. Estrogen stimulates the activity of the renin-angiotensin-aldosterone system, which is the main cause of water retention. A decrease in peripheral vascular resistance results from the effect of many active substances decreasing the vascular wall tone, including pregnancy hormones: estrogen, prolactin, placental lactogen, prostaglandin, or nitric oxide. The circulating blood volume increases from week 5-6 of pregnancy, most rapidly from weeks 20 to 24, while during the second half of pregnancy it still increases but in a considerably lower degree. The mean increase in blood volume is approximately 45%; however, high individual differences occur. The peripheral resistance decreases from the first week of pregnancy. Vascular resistance drops by approximately 10% in the first trimester of pregnancy; subsequently, its values are 35% lower from the initial values in week 20 of pregnancy; it remains constant by week 32 and then slightly increases [2, 40].

As a result of the effect on the haemodynamics of the cardiovascular system in pregnancy, the effect of a decrease in vascular resistance prevails over the effect of an increase in blood volume, resulting in a decrease in systolic and diastolic pressure. Decreased vascular resistance may result in an increased blood flow through many organs, for example, the uterus, kidneys, and skin [2].

It is assumed that the choroid, as a tissue built mainly of blood vessels, in which the blood flow constitutes 85% of the blood flow through the eyeball, should respond by an increase in its thickness to an increase in blood flow and drop in the value of peripheral resistance. Some researchers have explained the increase in the choroidal thickness in pregnant women observed in their studies based on this assumption [9, 21, 24, 25].

Blood flow through the blood vessels depends on perfusion pressure, and ocular perfusion pressure (OPP) is considered to be a driving force of the blood circulation in the eye [41, 42]. The study conducted by Kim et al. showed a statistically significant relationship between subfoveal choroidal thickness and OPP in the examined young healthy individuals [10]. In available reports concerning studies on the choroidal thickness in pregnant women, the relationship between choroidal thickness and OPP has been assessed in several studies [9, 21, 22, 24, 25]. Despite the fact that in all these studies a significantly higher statistically choroidal thickness was observed in pregnant women, only the study by Sayin et al. found a positive relationship between the OPP and choroidal thickness in pregnant women [24].

There is a lack of research which would trace choroidal thickness in the same women in all trimesters of pregnancy, which would allow an evaluation of whether the changes taking place in the third trimester, such as volume overload which is the highest in the third trimester, are transposed into the choroidal thickness. Dadaci et al. observed a decrease in the choroidal thickness in the third trimester of pregnancy. The researcher explained such a result by, among others, the fact that in the third trimester fluid volume increases by nearly 40%, and this decrease in choroidal thickness may result from the redistribution of blood to the uterus, skin, and kidneys [23]. In the study by Goktas et al., a significantly higher statistically choroidal thickness was observed in the second trimester. According to the researchers, the reduction of arterial pressure and vascular resistance, which occurs in the second trimester, is responsible for the result of the study [25]. In the remaining studies, no relationship was confirmed between gestational age and choroidal thickness [9, 20–22, 25].

### 2.5. Choroidal Thickness and Central Serous Chorioretinopathy

While interpreting changes in the choroidal thickness during pregnancy it was also assumed that, in association with the frequent occurrence of central serous chorioretinopathy (CSC) during pregnancy, to which the period of pregnancy is predisposed, and during which the choroid is thicker, an increased choroidal thickness should be expected in pregnancy [26]. A strong relationship between CSC and pregnancy is well known. A retrospective clinical-control trial showed that pregnancy is an important risk factor of CSC [43]. It is presumed that an increase in the choroidal thickness in patients with CSC is caused by an excessive permeability of the vessels [44]. The occurrence of CSC was also associated with the effect of corticosteroids, which may affect choroidal function leading to changes on the level of free radicals, prostaglandin, and NO, an elevated level of which leads to autoregulation disorders, abnormal blood pressure in capillaries, and, consequently, an increased permeability of choroidal vessels [45, 46]. At the beginning of their study, Kim et al. also assumed that, in pregnancy, due to more frequently diagnosed CSC, the choroid may be thicker. The study by Kim et al. included healthy pregnant women, women with the diagnosis of preeclampsia, and nonpregnant women. The authors of the study did not find any statistically significant differences in choroidal thickness between pregnant and nonpregnant women; however, they found that in the case of women with preeclampsia the choroid was statistically significantly thicker. According to Kim et al., pregnancy itself is related to neither an increased permeability of the choroid nor its increased thickness, but rather an increased permeability is observed only in some women in response to the effect of some factors, such as increased fluid volume, dilation of the vessels, reduced osmotic pressure, or changes in the level of prostaglandins, which may lead to CSC in pregnant women [26]. Takahashi et al. conducted a study to compare choroidal thickness between pregnant and nonpregnant women and tried to evaluate the relationship between CSC and pregnancy. The study covered a group of 25 pregnant women in the third trimester, while the control group were 27 women who were not pregnant [20]. In this study, like the study by Kim et al., no statistically significant differences in the choroidal thickness were found between pregnant women and the control group, in any of the measured areas [20, 26]. Concerning the relationship between choroidal thickness and CSC Takahashi et al. did not find such a relationship [20].

## 3. Conclusions

The problematic question of whether choroidal thickness in women with uncomplicated pregnancy is subject to change, and what mechanisms may be responsible for these changes, requires further studies. The choroidal thickness is affected by many factors, which considerably hinder the assessment of what mechanisms affect the change of its thickness. This assessment is even more difficult because the mechanism of autoregulation in the choroidal blood flow has not been fully recognized. Considering the fact that the character of the EDI-OCT examination, which is precise, repeatable, quick, and noninvasive, has great potential in the recognition of the effect of various factors on the anatomy of the choroid. It would be advisable to consider it for further studies concerning choroidal thickness in pregnant women. These studies should concern women in all three trimesters of pregnancy and consider the factors exerting an effect on choroidal thickness which have already been recognized.

## Figures and Tables

**Table 1:** Characteristics of included studies.

Studies	Study groups with the numbers of included women				Age (yr) Mean ± SD Normal pregnant women	Age (yr) Mean ± SD Control group	Gestational age (wk)+ mean ± SD	EDI-OCT	Type of measurement	Measured areas	SFCT (um) Mean ± SD	
	Normal pregnant women (*n*)	Normal non-pregnant women (*n*)	Women with Preeclampsia (*n*)	Women after pregnancy (*n*)							Normal pregnant women	Control group
Liu et al.	46	71	Not applicable	Not applicable	27.87 ± 3.65	28.43 ± 3.15	28–38 (30.20 ± 1.63)	Heidelberg	Manually	SFCT, 1 mm and 3 mm nasally, temporally, superiorly, inferiorly	344.13 ± 50.94	315.03 ± 60.57

Takahashi et al.	25	27	Not applicable	Not applicable	30.4 ± 6.0	30.0 ± 6.3	27–38 (31.0 ± 3.2)	Heidelberg	Manually	SFCT, 3 mm temporally; 3 mm nasally; 3 mm superiorly; 3 mm inferiorly	275 ± 84	273 ± 92

Kara et al.	100	100	Not applicable	Not applicable	28.6 ± 6.3	30.5 ± 6.2	15–38 (27.3 ± 6.6)	Cirrus HD-OCT	Manually	SFCT	371.1 ± 61.8	337.2 ± 62.4

Ulusoy et al.	29	36	Not applicable	29	27.41 ± 5.48	28.00 ± 5.27	28–39 (31.97 ± 3.77)	Heidelberg	Manually	SFCT	387.97 ± 59.91	320.86 ± 59.18

Dadaci et al.	27	25	Not applicable	Not applicable	28.9 ± 5.5	29.2 ± 7.0	6–8 (6.8 ± 0.8) 32–37 (35.1 ± 1.9)	Cirrus HD-OCT	Manually	SFCT; three locations nasal and three locations temporal to the fovea at 500 um intervals	*I trimester OP* 349.22 ± 82.11 *μ*m *III trimester OP* 333.56 ± 76.61 *μ*m *I trimester OL* 341.30 ± 85.22 *μ*m *III trimester OL* 326.93 ± 75.84 *μ*m	*OP*-318.88–53.13 *OL*-310.60–51.09

Sayin et al.	46	40	33	Not applicable	28.1 ± 6.2	30.2 ± 6.2	17–37 (28.0 ± 5.8)	Cirrus-HD-OCT	Manually	SFCT	368.6 ± 67.6	334.8 ± 59.9

Goktas et al.	90: 30 - I trimester 30 - II trimester 30 – III trimester	30	Not applicable	Not applicable	28.5 ± 6.4 26.6 ± 4.2 26.9 ± 6.2	29.4 ± 2	7.4 ± 2.6 19.2 ± 2.9 33.1 ± 2.8	Heidelberg	Manually	SFCT; 3 mm temporally; 3 mm nasally	I trimester 362 ± 81 II trimester 395 ± 80 III trimester 368 ± 70	335 ± 86

Kim et al.	14	21	7	Not applicable	31.43 ± 2.88	30.43 ± 4.15	32.14 ± 2.69	Heidelberg	Manually	SFCT	274.23 ± 29.30	264.95 ± 21.03

Rothwell et al.	12	12	Not applicable	Not applicable	32.77 ± 2.08	32.92 ± 3.01	29–35 32.4	Heidelberg	Automatically	SFCT; + 9 subfields defined by the Early Treatment Diabetic Retinopathy Study (ETDRS)	319.58 ± 6.11	287.58 ± 43.44

Ataş et al.	25	26	27	Not applicable	27.76 ± 5.21	29 ± 7.1	31.37 ± 4.9	Heidelberg	Manually	SFCT	387.2 ± 60.76	322.35 ± 63.89

**Table 2:** Association between subfoveal choroidal thickness with other factors in the study groups.

Studies	Study groups	AL	CCT	Age	Gestational age	DPB	SBP	MBP	OPP	BCVA	RE	IOP
Liu et al.	Pregnant women	A	C	A	C	C	C	B	C	B	C	C
	Nonpregnant women	A	C	A	D	C	C	B	C	B	C	C

Takahashi et al.	Pregnant women	A	B	C	C	C	C	B	B	B	A	C
	Nonpregnant women	A	B	C	D	C	C	B	B	B	A	C

Kara et al.	Pregnant women	C	C	C	C	B	B	B	C	B	C	C
	Nonpregnant women	B	B	B	D	B	B	B	B	B	B	B

Dadaci et al.	Pregnant women	B	B	B	A	B	B	B	B	B	B	B
	Nonpregnant women	B	B	B	D	B	B	B	B	B	B	B

Ulusoy et al.	Pregnant women	C	B	C	C	B	B	C	C	C	B	C
	Nonpregnant women	C	B	C	D	B	B	C	C	C	B	C
	Women after pregnancy	C	B	C	D	B	B	C	C	C	B	C

Sayin et al.	Pregnant women	C	C	C	A	B	B	B	A	B	C	C
	Nonpregnant women	C	C	C	D	B	B	B	C	B	C	C
	Women with Preeclampsia	C	A	C	C	B	B	B	C	B	C	C

Goktas et al.	Pregnant women	B	B	B	C	B	B	B	C	B	B	B
	Nonpregnant women	B	B	B	D	B	B	B	C	B	B	B

Kim et al.	Pregnant women	B	B	B	B	B	B	B	B	B	B	B
	Nonpregnant women	B	B	B	D	B	B	B	B	B	B	B
	Women with Preeclampsia	B	B	B	B	B	B	B	B	B	B	B

Rothwell et al.	Pregnant women	B	B	B	B	B	B	B	B	B	B	B
	Nonpregnant women	B	B	B	D	B	B	B	B	B	B	B

Ataş et al.	Pregnant women	B	B	B	B	B	B	B	B	B	B	B
	Nonpregnant women	B	B	B	D	B	B	B	B	B	B	B
	Women with Preeclampsia	B	B	B	B	B	B	B	B	B	B	B

A: statistically significant relationship; B: not rated; C: no dependency has been demonstrated; D: not applicable; Al: axial length; CCT: central corneal thickness; DBP: diastolic blood pressure; SBP: systolic blood pressure; MBP: mean blood pressure; OPP: ocular perfusion pressure; BCVA: best corrected visual acuity; RE: refractive error/spherical refraction; IOP: intraocular pressure.
